# Efficacy of *Helicobacter pylori* eradication regimens in Rwanda: a randomized controlled trial

**DOI:** 10.1186/s12876-018-0863-2

**Published:** 2018-08-30

**Authors:** Jean Damascene Kabakambira, Celestin Hategeka, Cameron Page, Cyprien Ntirenganya, Vincent Dusabejambo, Jules Ndoli, Francois Ngabonziza, DeVon Hale, Claude Bayingana, Tim Walker

**Affiliations:** 1 Kigali University Teaching Hospital (CHUK), Kigali, Rwanda; 2 Centre for Health Services and Policy Research, School of Population and Public Health, Faculty of Medicine, University of British Columbia, Vancouver, BC Canada; 3 Collaboration for Outcomes Research and Evaluation, Faculty of Pharmaceutical Sciences, University of British Columbia, Vancouver, BC Canada; 4 Department of Medicine, University Hospital of Brooklyn, New York, USA; 5 Butare University Teaching Hospital (CHUB), Huye, Rwanda; 6 Department of Medicine, University of Utah School of Medicine, Salt Lake City, UT USA; 7 School of Medicine and Public Health, Faculty of Health and Medicine, University of Newcastle, Newcastle, Australia

**Keywords:** *H. pylori* eradication, Dyspepsia, Clinical trial, Rwanda

## Abstract

**Background:**

Successful *H. pylori* treatment requires the knowledge of local antimicrobial resistance. Data on the efficacy of *H. pylori* eradication regimens available in sub-Saharan Africa are scant, hence the optimal treatment is unknown.

Our goals were to determine the efficacy of available regimens in Rwanda as well as evaluate the effect of treatment on health-related quality of life (HRQoL) in patients undergoing esophagogastroduodenoscopy**.**

**Methods:**

This is a randomized controlled trial conducted from November 2015 to October 2016 at a tertiary hospital in Rwanda. Enrollees were 299 patients (35% male, age 42 ± 16 years (mean ± SD)) who had a positive modified rapid urease test on endoscopic biopsies. After a fecal antigen test (FAT) and HRQoL assessment by the Short Form Nepean Dyspepsia Index (SF-NDI) questionnaire, patients were randomized 1:1:1:1 to either a triple therapy combining omeprazole, amoxicillin and one of clarithromycin/ciprofloxacin/metronidazole or a quadruple therapy combining omeprazole, amoxicillin, ciprofloxacin and doxycycline. All therapies were given for a duration of 10 days. The outcome measures were the persistence of positive FAT (treatment failure) 4 to 6 weeks after treatment and change in HRQoL scores.

**Results:**

The treatment success rate was 80% in the total population and 78% in patients with a history of prior triple therapy. Significant improvement in HRQoL in the total group (HRQoL mean scores before and after treatment respectively: 76 ± 11 and 32 ± 11, *p* < 0.001) and the group with functional dyspepsia (HRQoL mean scores before and after treatment respectively: 73 ± 11 and 30 ± 9, *P* < 0.001) was observed across all treatment groups.

Using clarithromycin based triple therapy (standard of care) as a reference, the group treated with metronidazole had worse HRQoL (*p* = 0.012) and had a trend towards worse treatment outcome (*p* = 0.086) compared to the ciprofloxacin based combination therapies.

**Conclusion:**

Clarithromycin and ciprofloxacin based combination therapies are effective and safe to use alternatively for *H. pylori* eradication and improve HRQoL. Among the regimens studied, metronidazole based triple therapy is likely to be clinically inferior.

**Trial registration:**

The clinical trial was retrospectively registered (PACTR201804003257400) with the Pan African Clinical Trial Registry database, on April 6th, 2018 in South Africa.

## Background

*Helicobacter pylori* is a known successful human pathogen living on the luminal surface of the gastric epithelium, responsible for various gastro-intestinal pathologies. The prevalence of infection is greatest in countries of the developing world. The most recent Rwandan *H. pylori* prevalence study in 2012 found a prevalence of 75% in an endoscopy population in Southern Rwanda [1]. The prevalence was the same three decades earlier in an endoscopy population in Kigali City, Rwanda [2]. For successful eradication policies in any country, there is need for accurate diagnostic tests and treatment tailored to local antibiotic resistance patterns.

### *H. pylori* diagnosis

There is no single gold standard diagnostic test for *H. pylori*. The choice of a diagnostic test is influenced by the pretest probability of infection, cost, availability, population prevalence of infection and factors such as the previous use of proton pump inhibitors (PPIs) and antibiotics that may alter test results. Several diagnostic tests rely on testing of endoscopy biopsy samples, serum or stools. The urea breath test uses a different technique based on the principle that *H. pylori* hydrolyses urea to produce carbon dioxide and ammonia but it is a nuclear medical technique that requires the use of carbon 13 and 14 isotopes that are not currently available in Rwanda. Major diagnostic challenges exist when it comes to checking eradication after treatment in resource-poor settings. The urea breath test which is the best option to document eradication is not available in most resource limited countries. The Maastricht V/Florence Consensus also suggests bacterial culture for antibiotic resistance testing in those who fail first line therapy [3]. However, *H. pylori* has historically been difficult to culture although techniques are improving. It is also known that *H. pylori* antibiotic sensitivity in vitro may not always predict response in vivo [4]*.* No laboratory in Rwanda cultures *H. pylori* reliably at present, and resistance testing is also not available.

Fecal antigen assays have been reported in the literature to have sensitivity and specificity above 90% [4–6].

### *H. pylori* treatment

Several studies have been conducted to evaluate efficacy of available *H. pylori* infection combination treatments [7–9]. The common practice is a combination of a PPI and two antibiotics (triple therapy) or the addition of bismuth salts to these three drug agents (quadruple therapy). The choice of antibiotics should be region specific, based on local *H. pylori* resistance to those antibiotics. However, raising antimicrobial therapy resistance to *H. pylori* poses great management challenge worldwide. In Korea, Byoungrak et al. investigated antibiotic resistance in isolates in two cohorts in 2009–2010 and 2011–2012. Resistance to metronidazole was found to be 45.1% and 56.3% respectively in those two cohorts [10]. In Brazil, Eisig et al. detected *H. pylori* resistance to metronidazole and clarithromycin of 51% and 8% respectively [11]. In Africa, resistance to metronidazole is common. In Cameroon, *H. pylori* resistance was found to be as high as 93% for metronidazole, 85% for amoxicillin, 45% for clarithromycin and 44% for tetracycline in 2008 [12]. In South Africa, marked resistance (96%) to metronidazole was observed. However marked susceptibility to ciprofloxacin (100%), amoxicillin (98%), clarithromycin (80%) and gentamicin (73%) was observed in the same study [13].

No prior studies have assessed *H. pylori* antibiotic resistance in Rwanda. However, data suggest that more than half of patients presenting for endoscopy with a history of prior triple therapy remain positive for *H. pylori* infection, raising concern about potentially high resistance rates to current triple therapies used for *H. pylori* eradication [1]. According to the Rwandan Internal Medicine Clinical Treatment Guidelines, the first choice for *H. pylori* eradication regimen is a triple therapy combining omeprazole 20 mg twice daily, clarithromycin 500 mg twice daily and amoxicillin 1 g twice daily for a 10–14 day duration [14]. While the susceptibility of *H. pylori* to clarithromycin varies widely from 55 to 94% across the world, the efficacy of the clarithromycin-based triple therapy in Rwanda is unknown. Furthermore, the cost of clarithromycin-based regimen is approximately US$18, making it less affordable in a country with a GDP per capita of US$703 and an annual total health spending per capita of US$ 55 [15]. The present study assessed the efficacy of other cheaper *H. pylori* eradication regimens, pragmatically constructed using antibiotics cheaply available in Rwanda.

### Health related quality of life

Functional dyspepsia is defined as the presence of one or more of the following: epigastric pain or burning, postprandial fullness and early satiety without evidence of structural disease (including at upper endoscopy) that can explain the symptoms [16]. Whether *H. pylori* improves quality of life among patients with functional dyspepsia, remains controversial. A study by Lane et al. in 2006 failed to detect an improvement in HRQoL after *H. pylori* eradication [17]. A large meta-analysis by Li-Jun revealed contradictory results across 25 randomized controlled trials and recommends individual assessment for clinicians desiring to eradicate *H. pylori* in patients with functional dyspepsia [18]. The American College of Gastroenterology acknowledges the lack of sufficient evidence to show the benefit of treating *H. pylori* in patients with functional dyspepsia but currently recommends testing for and treating *H. pylori* given durable benefit documented in some patients in previous studies [19–21].

The current study examines the efficacy of ciprofloxacin and metronidazole based *H. pylori* eradication therapies compared with clarithromycin based triple therapy, and the observed impact on HRQoL, among patients referred for endoscopy.

## Methods

### Patients

The study enrolled patients attending the University Teaching Hospital of Butare (CHUB) for esophagogastroduodenoscopy (EGD). CHUB is one of the 4 tertiary level hospitals in Rwanda with approximately 380 beds, located in the Southern Province of Rwanda. Patients are referred for EGD at CHUB by clinicians working in inpatient and outpatient facilities of CHUB, as well as satellite district hospitals and private health facilities in the town. Endoscopies are undertaken by two gastroenterologists or by a trainee physician under the supervision of the gastroenterologists.

The study enrolled patients who were 21 years and older, had a positive modified rapid urease test on endoscopic biopsies and were willing to come back for follow-up. Patients were excluded from the study if they: had used PPIs or a histamine H2 receptor antagonist or antimicrobial therapy in the previous 4 weeks; were allergic to any of the study drugs (omeprazole, amoxicillin, clarithromycin, ciprofloxacin, metronidazole and doxycycline); or had endoscopic or clinical evidence of gastric malignancy. Female patients were not breastfeeding and had a negative pregnancy test prior to randomization.

### Randomization and study process

The study required two visits to CHUB, in Huye District in the Southern Province of Rwanda.

At visit 1, patients underwent endoscopy. Biopsies were tested for *H. pylori* infection by the MRU test. Patients provided a stool sample for fecal antigen test and completed a questionnaire about medical history as well as the HRQoL questionnaire. Randomization was done by picking a folded piece of paper under the observation of a study nurse, from a basket containing thoroughly mixed pieces of paper labeled with numbers corresponding to one of the 4 arms of treatment, each in equal quantity.

Patients and the treating clinicians were blinded to treatment. However, given that a high number of patients were unable to read and comprehend drug dosage instructions in English, a different nurse not associated with study analysis, opened envelopes containing medications together with the patients to explain how to take them. This nurse was not allowed to discuss this information or treatment allocations with the treating clinicians or study staff; neither was she allowed to complete patient assessments at the second visit.

At visit 2 scheduled at 4–6 weeks after treatment completion, patients were clinically evaluated by a study clinician and completed the HRQoL questionnaire again. Patients with an initially positive fecal antigen test (FAT) also provided a stool sample for a post-treatment FAT.

### Investigations

*H. pylori* status was determined by urease activity on 4 (2 antral and 2 fundal) biopsies. MRU test was undertaken by exposing gastric antral/body biopsies to a solution of 1 ml of 10% urea in water to which a drop of 1% phenol red has been added. When *H. pylori* is present, the bacterial urease catalyzes urea to ammonia and carbon dioxide which can be detected by the typical red color change in the solution [22].

A positive reaction, manifested by a color change within 3 h, was necessary for patients to be eligible for the study.

Patients were also required to provide stool samples for FAT. Patients who were unable to provide samples the same day were instructed to return samples the following morning before starting medication. FAT was performed with a rapid antigen test (HEALGEN;ORIENT GENE;DS; catalog number GCHP-602).

### Treatment regimens

Enrollees were randomized to one of the four following regimens:

- Group 1: omeprazole 20 mg twice daily + amoxicillin 1 g twice daily + clarithromycin 500 mg twice daily for 10 days (CLARITHRO).
- Group 2: omeprazole 20 mg twice daily + amoxicillin 1 g twice daily + ciprofloxacin 500 mg twice daily for 10 days (CIPRO).
- Group 3: omeprazole 20 mg twice daily + amoxicillin 1 g twice daily + metronidazole 500 mg three times a day for 10 days (METRO).
- Group 4: omeprazole 20 mg twice daily +amoxicillin 1 g twice daily + ciprofloxacin 500 mg twice daily+ doxycycline 100 mg twice daily for 10 days (CIPRO-Plus).

### Health related quality of life (HRQoL)

During the first and second visits, the Short Form Nepean Dyspepsia Index (SF-NDI) questionnaire was completed to assess HRQoL before and after treatment.

The SF-NDI questionnaire has been translated and validated for use in a Kinyarwanda speaking population [23]. SF-NDI is a questionnaire with 10 items measured on six-point Likert scales. The instrument assesses five domains namely: tension/anxiety, interference with daily activities, knowledge/control, eating/drinking and work/study. Individual items in each sub-scale are aggregated to obtain a score range from 0 (best HRQoL score) to 100 (worst HRQoL score) as defined by the questionnaire developers [24].Questionnaires were available in English and Kinyarwanda. Adapted questionnaire was availed for illiterate patients to be completed with the help of a literate support person.

### *H. pylori* eradication

Patients with initially positive FAT had a second FAT performed at visit 2. Patients were considered *H. pylori* negative if the second FAT was negative.

### Safety considerations

Patients were instructed to report any side effects through a phone hotline to the research group. Symptoms were classified as mild, moderate or severe. Patients with severe symptoms were instructed to cease medication and were assessed by a gastroenterologist who decided on a further treatment regimen. At visit 2, a follow-up with a gastroenterologist was arranged for all patients who were still symptomatic.

### Study outcomes

#### Primary outcome

- Positive fecal antigen test 4 to 6 weeks after treatment (among those with an initially positive fecal antigen test).

#### Secondary outcome

- Health related quality of life score change by SF-NDI questionnaire.

### Statistical analyses

An intention-to-treat analysis was planned and conducted. Data are presented as mean ± standard deviation (SD) for normally distributed continuous variables and frequencies (percentage) for categorical variables. To compare the efficacy of each treatment arm vs standard of care (i.e., CLARITHRO), we performed independent samples t-test for continuous outcome (i.e., mean change in HRQoL score) and Fisher exact test for categorical outcomes. A paired t-test was used to compare pre- and post-treatment HRQoL scores. Statistical analyses were two-tailed and *p* values of < 0.05 were considered to show statistical significance. Analyses were performed with STATA (v 15, College Station, Texas).

## Results

In a period of 1 year, from November 2015 to October 2016, a total of 866 patients underwent EGD. Of the 866 patients, 308 had a negative modified rapid urease (MRU) test for *H. pylori* while 96 patients did not undergo MRU testing.

**Fig. 1:**
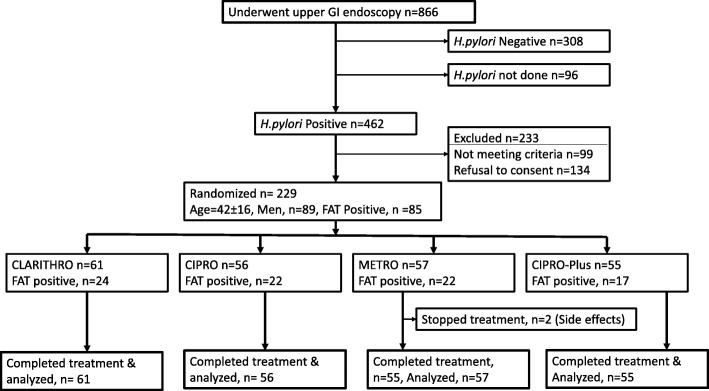
Flow chart for patient selection and randomization

Therefore, 462 patients had a positive MRU test but 99 patients did not meet inclusion criteria while 134 patients declined to participate to the study. Thus, 229 patients (35% male, age 42 ± 16 (mean ± SD), range 21-81 years) were randomized (Fig.1).

### Baseline characteristics

The patient characteristics of the overall study group and each assigned treatment group are presented in Table 1. Access to EGD was generally rapid (mean number of days between endoscopy request by treating physician and endoscopy day: mean 2.2 ± 3.1).The population had a female preponderance at 65%. All patients carried a health insurance: 88% possessed community health insurance (Mutuel de Santé) while 12% had private health insurance. Previous medication use was very common among the study population: 77% had used PPIs or histamine (H2) receptor antagonists, 17% had taken triple therapy before and 14% reported using antibiotics for other illnesses.

**Table 1:** Baseline population characteristics according to arm of treatment

Characteristics		Total (*n* = 229)	CLARITHRO *n* = 6127%	CIPRO *n* = 5624%	METRO n = 5725%	CIPRO-Plus *n* = 5524%
Demographic characteristics	Male (%)	35	43	43	35	35
	Age, years mean ± SD	42 ± 16	40 ± 15	44 ± 16	41 ± 16	41 ± 17
	Married (%)	57	52	68	60	49
	CommunityHealth Insurance (%)	88	85	88	93	87
	Access to endoscopy (days)	2.2 ± 3.1	1.9 ± 0.6	2.8 ± 6	2.3 ± 1.7	1.9 ± 0.6
Medical history	PPI or H2 blocker before (%)	77	74	79	79	78
	Triple therapy before (%)	17	13	23	16	18
	Antibiotics before (%)	14	13	13	13	18
Symptoms	Epigastric pain (%)	96	97	95	95	96
	Vomiting (%)	30	27	38	19	35
	Hematemesis (%)	8	10	9	4	9
	Melena (%)	2	2	4	2	2
Endoscopy finding	Normal endoscopy (%)	16	18	14	21	11
	Gastritis (%)	56	41	55	61	67
	Gastric ulcer (%)	11	10	14	14	7
	Duodenal ulcer (%)	30	41	36	25	16
Initially positive FAT (%)		37	39	39	39	31
Baseline HRQoLtotal group(mean ± SD score)		76 ± 11	77 ± 13	76 ± 12	76 ± 8	78 ± 12
Baseline HRQoLin functional dyspepsia (*n* = 37) (mean ± SD score)		73 ± 12	71 ± 9	70 ± 17	74 ± 10	76 ± 14

Baseline characteristics of the intention to treat population were not significantly different, except for gastritis (CIPRO Plus and METRO were significantly different from CLARITHRO, with *p* = 0.005 and *p* = 0.02, respectively) and for duodenal ulcer (CIPRO Plus was significantly different from CLARITHRO, *p* = 0.004).Abbreviations: *SD* Standard deviation, *FAT* Fecal Antigen Test, *PP* Proton Pump Inhibitors

The most common presenting symptoms were epigastric pain (96%) and vomiting (30%).

The most common endoscopic diagnoses were gastritis (56%), duodenal ulcer (30%) and gastric ulcers (11%). There was no lesion seen on endoscopy in 37 (16%) patients. These patients with functional dyspepsia had lower baseline HRQoL score compared to the patients with lesions on endoscopy (73 ± 12 and 77 ± 11, *p* = 0.027). Therefore, a post-hoc subgroup analysis was performed after treatment with regards to HRQoL. *H. pylori* testing at endoscopy was positive in 60% of patients who underwent the modified rapid urease test. Only 37% (85/229) patients had a positive FAT. The mean HRQoL score was 76 ± 11 and there was no sex difference in all subdomains, thus men and women were analyzed together after treatment. The previous use of PPIs or H2 blockers was neither associated with negativity of the FAT (OR = 1.1, *p* = 0.847 CI [0.5,2.2]) nor difference in HRQoL among users versus non-users (76 ± 11 and 77 ± 12, *p* = 0.354). Similarly, a previous exposure to triple therapy was neither associated with negativity of the FAT (OR = 1.1, *p* = 0.738 CI [0.5, 2.7]) nor difference in HRQoL among users versus non-users (75 ± 12 and 77 ± 11, *p* = 0.549).

Overall, there was no difference in baseline characteristics with regards to prior exposure to triple therapy except for the presence of duodenal ulcers that were more common among the non-exposed than in the exposed group (70% vs 30%, *p* = 0.025). Therefore, a subgroup analysis was undertaken with regards to prior exposure to triple therapy status.

### Efficacy of *H. pylori* eradication regimens

#### Treatment failure

Of the total 85 patients who had an initially positive FAT, 20% (17/85) had a positive test after treatment. Compared to the treatment success in the overall group, there was a trend towards higher treatment failure in the METRO group although this did not reach statistical significance (36%, *p* = 0.086) (Table 2). Analysis stratified to the group without prior exposure to triple therapy showed a similar trend in treatment failure with the highest proportion in the METRO group (39%, *p* = 0.140).The sample size of the group with prior exposure to triple therapy was too small to establish a statistically sound comparison.

**Table 2:** Study outcomes

Outcome		Total *N* = 229	CLARITHRO *n* = 6127%	CIPRO *n* = 5624%	METRO *n* = 5725%	CIPRO-Plus *n* = 5524%	*p*-value^1^
Treatment failure (Positive FAT) (%)	Total group (*n* = 85)	20	13	18	36	12	0.191
	No prior exposure to triple therapy (*n* = 73)	22	14	21	39	14	0.265
HRQoLin total group (mean ± SD)	Score after treatment	32 ± 11	31 ± 10	31 ± 10	36 ± 12**	31 ± 9	0.023
	Mean difference	44 ± 14	46 ± 15	44 ± 15	40 ± 13*	47 ± 13	0.032
HRQoL in functional dyspepsia (*n* = 37) (mean ± SD)	Score after treatment	30 ± 9	33 ± 13	28 ± 7	32 ± 6	24 ± 5	0.229
	Mean difference	42 ± 15	38 ± 17	42 ± 17	42 ± 10	51 ± 16	0.392
Persistence of symptoms (%)	Total group (*n* = 229)	22	18	23	26	20	0.715
	No prior exposure to triple therapy (*n* = 189)	23	19	26	29	18	0.492

^*^P-value by multiple regression for comparison to the reference group (CLARITHRO), *P ≤ 0.05, **P ≤ 0.01

^1^Student t-test or chi square test as appropriate

#### Health-related quality of life

**Fig. 2:**
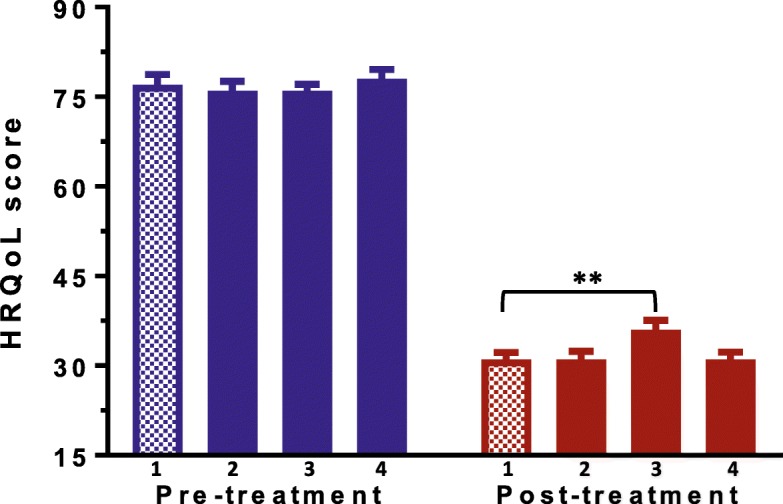
Health-Related Quality of Life scores before (blue) and 4 to 6 weeks after (red) treatment. Scores were measured by the use of the SF-NDI questionnaire on a range from 0 (best HRQoL) to 100 (worst HRQoL). Arms of treatment: 1: CLARITHRO, 2: CIPRO, 3: METRO, 4: CIPRO-Plus. ^*^p-value by multiple regression for comparison to the reference (REF) group (CLARITHRO), *p ≤ 0.05, **P ≤ 0.01, ***P ≤ 0.001

There was a dramatic change in HRQoL scores from 76 ± 11 to 32 ± 11 after treatment (*p* = 0.032) in the total group. A paired t-test showed a significant improvement in HRQoL across all the four arms of treatment (*p* < 0.001) but a group comparison to the standard of care showed a significant difference only with the METRO group (Fig. 2). Infact, the METRO group maintained a higher post-treatment score than the standard of care (36 ± 12 vs 31 ± 10, *p* = 0.008) and registered a lower mean score change than the standard of care (40 ± 13 vs 46 ± 15, *p* = 0.012). In the group with functional dyspepsia, a paired t-test showed significant improvements across all the four arms of treatment. Further analysis revealed that improvement in HRQoL score was not different in all the four arms of treatment compared to the standard of care (Table 2).

An assessment of clinical evolution after treatment revealed a persistence of symptoms in 22% of the total population. The METRO group had the highest rate of persistence of symptoms (26%) followed by CIPRO (23%), CIPRO-Plus (20%) and CLARITHRO (18%) but no regimen was statistically different from the standard of care.

Patients with persistence of symptoms after treatment had a higher HRQoL than patients who experienced symptom resolution (35 ± 11 vs 31 ± 10, *p* = 0.018) suggesting that persistence of symptoms was associated with worse HRQoL. Stratified analysis within the group of patients with functional dyspepsia (*n* = 37) showed a significant change in HRQoL scores overall (mean difference score: 42 ± 15, *p* < 0.001) but group comparison to the standard of care revealed no difference in any arm of treatment. The greatest improvement in HRQoL was observed in “interference with daily activities” (*p* = 0.019) and “eating/drinking” (*p* = 0.050) subdomains.

#### Drug safety and specific treatment related side effects

A total of 34% (79/229) patients reported treatment related adverse effects. The adverse effects were reported to the investigator and were classified as mild, moderate or severe. The most commonly reported side effects were taste perversion (27%), nausea (18%), dizziness (14%) and vomiting (9%). Some patients had more than one adverse effect. Only two patients in the METRO group had symptoms severe enough to stop medication. These two patients were called back to see a gastroenterologist for further management but they were analyzed in the METRO group. Overall, 99% of patients completed treatment and no patient was lost to follow-up.

## Discussion

This study explored the efficacy of pragmatic *H. pylori* eradication regimens available in Rwanda. Our results indicate that treatment success rate was 80% in the total group and 78% in the group without prior exposure to triple therapy. Significant improvement in HRQoL, expressed by decrease in SF-NDI scores, from baseline was observed across all the 4 arms of treatment. Our study results align with eradication success rates found in other studies around the world. The Maastricht IV/ Florence Consensus Report reported that the widely used triple therapy regimen cured 70% of patients [25].

Although there was no statistically significant difference between any of the arms of treatment and the standard of care, the metronidazole (METRO) based triple therapy showed some signals to suggest metronidazole based triple therapy might be inferior to other regimens. Infact, metronidazole based triple therapy registered the highest failure rate (36%) followed by CIPRO (18%), CLARITHRO (13%) and CIPRO-Plus (12%).

Similarly, HRQoL was improved in all the treatment groups but improvement was much less in the metronidazole based triple therapy than in the standard of care. Although the metronidazole based triple therapy was inferior to CLARITHRO group in improving HRQoL, the difference in treatment failure did not reach the level of statistical significance, probably due to lower than expected failure rates overall, and the concomitant reduction in study power. No susceptibility study has ever been conducted particularly in Rwanda but the resistance of *H. pylori* to metronidazole is notoriously high (90–100%) in Africa, which may explain the modest performance of metronidazole based triple therapy that was found [26–28].

On the other hand, ciprofloxacin based triple therapy (omeprazole + amoxicillin+ ciprofloxacin) and quadruple therapy (omeprazole + amoxicillin+ ciprofloxacin+ doxycycline) were not inferior to clarithromycin based triple therapy and presented a very good safety profile. This finding aligns nicely with studies conducted in Nigeria and South Africa which didn’t detect any resistance to ciprofloxacin, suggesting that a fluoroquinolone based regimen may be of utility in Africa [12, 27]. Pending more rigorous diagnostic tests for eradication, this finding offers hope that the combination of omeprazole, amoxicillin, ciprofloxacin and doxycycline could be used as a salvage therapy, particularly since bismuth combinations are still unaffordable in Rwanda.

This study shows that *H. pylori* was positive in 60% of the eligible population. This prevalence is lower than other prevalences reported in Africa possibly due to the fact that a portion of our population had previously been treated for *H. pylori* [28].

Although reported to be of high accuracy for initial and post-treatment diagnosis, FAT was only able to detect 37% of patients with *H. pylori.*

The diagnostic performance of FAT shows large variations across the world. Studies assessing the diagnostic accuracy of FAT have concluded to sensitivities and specificities above 90% in Europe and Taiwan [4–6]. A study conducted in Uganda, a neighboring country with Rwanda, found a contrasting sensitivity and specificity of 56% and 74% respectively [29]. In Nigeria, Olufemi et al. reported *H. pylori* prevalence of 68.7% using a serology test but it was only 20.2% using FAT [30]. The poor performance of FAT in this study as well as the two studies in Uganda and Nigeria raise concern about the utility of FAT as a diagnostic test in Rwanda and in Africa in general. Evidence shows that prior exposure to PPIs interferes with diagnostic accuracy of FAT but we had attempted to control this problem by excluding patients who used PPIs or histamine receptor antagonists in the past 4 weeks [31]. The explanation of the diversity in FAT accuracy as a test which uses antigens is most likely to be linked with the diversity of genome and virulence of *H. pylori* strains across the world [32]. Emerging data from studies predominantly conducted in Asian populations unequivocally show large geographical variations in the distribution of *H. pylori* strains [33, 34]. Although extensive work has been done to elucidate how genetic diversity is related to human cancer, little is known about the effect of genetic diversity on the performance of stool antigen test for the diagnosis of *H. pylori* [35]*.*

Our study found significant change in HRQoL scores from baseline across all the 4 arms of treatment. The findings were even true in the sub-group of patients who had normal endoscopy before treatment (functional dyspepsia).

This finding adds to the currently accumulating literature in favor of improvement of HRQoL by treating *H. pylori* in patients with functional dyspepsia. However, we had a small number of patients with functional dyspepsia and we are not statistically powered enough to draw a firm conclusion. Further studies with larger sample sizes are still needed to confirm this finding.

Lastly, although all study participants had health insurance, most (88%) had community health insurance, which is the cheapest and usually the main option accessible to Rwandans with limited financial resources. Community health insurance only gives access to health care and medicine available in public health care facilities. Due to the high cost and low availability in many public facilities, clarithromycin is the least affordable medication for the great majority of patients. It is not available at all in many rural health care facilities. Because of this, clinicians struggle to select appropriate initial and salvage regimens for *H. pylori* eradication. The treatment success rate trend and safety profile from this study make the ciprofloxacin based combination therapies a strong and cost-effective alternative to clarithromycin based therapy.

### Strengths and limitations

This is the first ever *H. pylori* eradication clinical trial conducted in Rwanda. By using a multi-arm trial, we were able to study efficacy of various *H. pylori* eradication regimens simultaneously using the same control group, thus saving time and resources to provide locally appropriate evidence to guide clinical practice. Findings are important because they offer chance to clinicians to prescribe affordable treatment regimens with confidence.

Due to financial constraints, this study was conducted in one center and the outcome measurement was limited to the use of FAT, which turned out to be a poorly sensitive diagnostic test in our study population, thus limiting the study’s power.

The treatment duration was 10 days across all the four arms of treatment in our study. Infact, earlier studies did not report major differences between short duration treatments (7 days) and long duration treatments (14 days) [36]. Our choice of a 10 day duration was inspired by the Maastricht IV/ Florence Consensus Report which suggested that extending therapies to 10–14 days improves eradication success by 5% [25]. We chose the lower end of the optimal duration due to financial and adverse drug effect considerations. It is important to mention that the weight of recent literature now advocates a long course of treatment (14 days) to optimize outcomes [21, 37].

This study raises awareness in policy makers and paves a path for subsequent studies that will apply more rigorous diagnostic methods such as bacterial culture and urea breath test to better characterize *H. pylori* eradication in sub-Saharan Africa. Further work needs to be done examining other alternatives, including high dose dual therapy, if treatment recommendations are to be optimized.

## Conclusion

Given the balance of cost, efficacy and safety profile documented in this study; clinicians should feel confident to use clarithromycin and ciprofloxacin based combination therapies for *H. pylori* eradication in Rwanda. Our findings suggest that metronidazole based triple therapy is likely to be clinically inferior, and make it the worst choice among the four regimens we studied.

## Abbreviations

CHUB: Butare University Teaching Hospital
CHUK: Kigali University Teaching Hospital
CIPRO: Omeprazole 20 mg twice daily + amoxicillin 1 g twice daily + ciprofloxacin 500 mg twice daily for 10 days
CIPRO-Plus: Omeprazole 20 mg twice daily + amoxicillin 1 g twice daily+ ciprofloxacin 500 mg twice daily+ doxycycline 100 mg twice daily for 10 days
CLARITHRO: Omeprazole 20 mg twice daily + amoxicillin 1 g twice daily + clarithromycin 500 mg twice daily for 10 days
EGD: Esophagogastroduodenoscopy
FAT: Fecal Antigen Test
GDP: Gross domestic product
HRQoL: Health-related quality of life
METRO: Omeprazole 20 mg twice daily + amoxicillin 1 g twice daily + metronidazole 500 mg three times a day for 10 days
MRU: Modified rapid urease
PPI: Proton Pump Inhibitors
SD: Standard deviation
SF-NDI: Short Form Nepean Dyspepsia Index
